# First report of *Rhabditis* (*Rhabditella*) *axei* with the invasive palm borer *Paysandisia archon*

**DOI:** 10.2478/jofnem-2024-0005

**Published:** 2024-03-14

**Authors:** Chiara Sciandra, Sara Amoriello, Emilia Innocenti Degli, Valentina Nicotera, Francesco Barbieri, Giuseppe Mazza, Giulia Torrini, Pio Federico Roversi, Agostino Strangi

**Affiliations:** Consiglio per la Ricerca in agricoltura e l'analisi dell'economia agraria – Centro di Ricerca Difesa e Certificazione, Firenze, Italy; Università degli Studi di Siena, Dipartimento di Scienze della Vita, Siena, Italy; National Biodiversity Future Center, Palermo 90133, Italy

**Keywords:** Alien invasive species, Italy, Lepidoptera, nematode-insect interaction, Rhabditidae

## Abstract

*Rhabditis (Rhabditella) axei* is a free-living, pseudoparasitic, necromenic, and parasitic nematode, depending on the host. This species feeds mainly on bacteria present in decaying organic matter, soil, and other substrates; however, in its parasitic form, it can colonize some species of snails. Moreover, the presence of *R. axei* has also been detected in birds and mammals, including humans.

In 2021–2023, during monitoring of the palm borer *Paysandisia archon* in Central Italy, *R. axei* emerged from dead larvae of this alien invasive moth and was extracted from palm fibres of *Trachycarpus fortunei* in three independent sites. The nematode was identified by morphological and morphometric analyses. Molecular analyses using SSU and LSU gene fragments were used to confirm the identification and to perform Bayesian reconstruction of the phylogeny. Each sampling site showed a unique haplotype.

Concerning the pathogenicity of this nematode against insects, the test performed on *Galleria mellonella* larvae did not show any entomopathogenic effect. This is the first time that *R. axei* was found associated with *P. archon,* and this recurrent association was discussed.

Rhabditidae is a heterogeneous family of nematodes often found in association with other organisms due to their necromenic-scavenger habits. In this ecological niche, nematodes feed primarily on bacteria. However, some *Rhabditis* species can switch from a free-living lifecycle to a pseudoparasitic or parasitic form, such as those of the subgenus *Rhabditella* (Mahmoud et al., 2019).

The genus *Rhabditella* was introduced by Cobb in 1929, but a more recent taxonomic revision considered *Rhabditella* as a *Rhabditis* subgenus (Sudhaus and Fitch, 2001). To date, the subgenus *Rhabditella* includes about seven species, most of which are the result of occasional isolations in the late twentieth century and were described only on the basis of morphological features (Sudhaus, 1991; Kiontke, 1999). Among *Rhabditella* subgenus, *Rhabditis (Rhabditella) axei* (Cobbold, 1884; Chitwood, 1933) is one of the most widely studied species. This organism was first described by Cobbold in 1884, but it was subsequently re-described twice by Chitwood (1933) and Goodey (1963) from different isolation sources.

Initially, *R. axei* was considered a ubiquitous saprophytic nematode that usually inhabits the soil and feeds on bacteria present in decaying matter. This classification was discussed when a medical case published by Feng and Li (1950) reported an episode of urinary infection in two patients in China; the possible risk for human health of this nematode was independently confirmed by Goldsmid (1967), who identified another case of urinary infection in a Zimbabwean woman. Since then, other cases of human infection in the gut or at the urinary apparatus have been detected in China and Iran in patients with a compromised immune system and/or poor access to clean water. However, such infections are speculated to be due to the presence of the nematodes in the urine or feces, and further studies are needed to verify the pathogenicity (Shao et al., 2003; Meamar et al., 2007; Shuo et al., 2018).

Moreover, *R. axei* has been identified in the feces of mammals such as dogs (Levine et al., 1963), the Asiatic brush-tailed porcupine *Atherurus macrourus* (Rakhshanpour et al., 2012), and chickens (El-Azazy et al., 1988). The relationship between the nematode and these hosts is considered pseudoparasitic (i.e., nematodes, mites, psocid, and pollen that are mistaken for parasites, usually introduced with food, without any relationship with the host animals; Pierre et al., 1995), and the findings were occasional events.

Several articles have reported the occurrence of *R. axei* in terrestrial snails and slugs of the genera *Achatina*, *Archachatina*, *Deroceras*, *Limax*, *Limicolaria,* and *Monacha* (Odaibo et al., 2000; Awharitoma and Edo-Taiwo, 2012; Olofintoye and Olorunniyi, 2016; d'Ovidio et al., 2019; Mahmoud et al., 2019; Brophy et al., 2020). The *R. axei* recovery in these snail genera is so common that it was also reported in specimens of the African *Ac. fulica* imported to Italy as pets (d'Ovidio et al., 2019). Although *R. axei's* presence in giant snails seems to be asymptomatic for the host, its presence in smaller snails like *M. cartusiana* leads to behavioral alterations such as reduced movement and food intake, as well as retraction inside the shell. Experimental data showed a quantitative correlation between increased mortality of *M. cartusiana* and exposure to different concentrations of *R. axei* infective juveniles (Mahmoud et al., 2019). However, Mahmoud et al. (2019) reported that *R. axei* leads a double life, ranging from free-living to necromenic in larger snails and parasitic behaviour in smaller snails. Such parasitic life was also reported by Awharitoma and Edo-Taiwo (2012), who found this nematode in the mantle, kidney, crop, and stomach of snails.

Unlike in mollusks, scanty data on the relationship between *R. axei* and insects are present. Hague (1963) reported that the presence of this nematode in a colony of *Stomoxys calcitrans* reared in the laboratory was able to reduce and delay the emergence of the fly, but the original source of nematode infestation was never traced, and the competition for substrate was hypothesized as an interaction model.

Although the presence of *R. axei* has been recorded in several taxa, as reported above, probably due to its distribution in several habitats, its role is not well understood and seems to be case-specific (references herein).

In Europe, there are few data on natural enemies or associated species of *P. archon*. In the literature, nematodes, mites, and fly larvae have been found on a dead *P. archon* larva in an insectarium, although it is most likely that these organisms simply colonized the larval body after death, which is a common occurrence. The nematode was identified as belonging to the genus *Rhabditis*, probably *Rhabditis* (*Choriorhabditis*) *longicaudatus* (Bastian, 1865) (i Monteys and Aguilar, 2005; Ortega-García et al., 2017).

During a monitoring project in Italy on the invasive palm borer *Paysandisia archon* (Lepidoptera: Castniidae) (Mori et al., 2022), we recorded a recurrent association between *R. axei* and this alien invasive moth. Here, we report and discuss this first association.

## Materials and Methods

### Sampling sites

During the periodic survey of *Trachycarpus fortunei* palms, to monitor the presence of the alien insect pest *Paysandisia archon* in Florence (Tuscany, Central Italy) and neighboring areas (Mori et al., 2022), four palms were found to be infested by this moth. The palm samples were collected in 1) November 2021, “Firenze-Isolotto” (43° 46′ N - 11° 12′ E); 2) December 2022, “Impruneta” (43° 42′ N - 11° 15′ E); 3) March 2023, “Firenze-Cascine del Riccio” (43° 44′ N – 11° 15′ E) and 4) December 2023, “Impruneta” (same location).

In the first palm sampled, located in an urban district of Florence, 15 *P. archon* larvae were found, five of which were dead. In the second palm, located outside Florence, eight dead larvae of the same insect were found. In the third infested palm, located in an area between the previous two, 25 dead *P. archon* larvae were collected. In the fourth palm, 43 larvae were found, 14 of which were dead. In addition, a mollusk of the species *Cornu aspersum* (Styllomathopora: Helicidae) was also found alive with the *P. archon* larvae (O.F. Müller, 1774).

### Nematode isolation

All dead *P. archon* larvae were rinsed in 2% sodium hypochlorite and individually placed in modified White traps (Kaya and Stock, 1997) to collect emerging nematodes. In addition, nematodes were extracted from palm fibers (200 cc for each palm) using the Oostenbrink dish method, which is widely used to extract nematodes from soil or litter samples (EPPO, 2013). Emerging nematodes were collected and incubated at 25±5 °C; 80–100% RH; under a natural light:dark (L:D) cycle of approximately 14:10.

The feces of all live larvae and mollusks collected from the fourth palm were analyzed to verify the presence of nematodes. In addition, four healthy *P. archon* larvae from the same sample were dissected (foregut, midgut and hindgut, malpighian tubules, fat bodies, and trachea) to verify the presence of nematodes *in situ*.

### Morphological and morphometric analyses

The extracted nematodes were mounted on temporary microscope slides as reported in Shurtleff and Averre (2000) and morpho-biometrically characterized. Specimens were observed under a light microscope Leica DM2000 equipped with MC 170 HD digital camera using LEICA Application Suite v. 4.9.0 as measuring software (Leica Microsystems, Heerbrugg, Switzerland). Morphological characters were selected according to Goodey (1963) and Meamar et al. (2007). The measures were taken from adult specimens (14 females and 21 males) collected from *P. archon* larvae of the second palm. The features were body length (L), maximum body diameter (MBD), buccal cavity length (B), tail length (T), and distance of the base of the pharynx from the head end (ES). In addition, distance of vulva from head end (V) and egg diameter (ED) were measured in females, while spicule length (SP) and gubernaculum length (GU) were measured in males. The following ratios were then calculated: a (L/MBD), b (L/ES), c (L/T), and V% (V/L*100).

### Scanning Electron Microscopy (SEM)

Live specimens of the nematode were hand-picked from the White trap with a small needle, transferred into a 1.5 ml microtube, and rinsed several times in tap water. Specimens were then fixed, dehydrated, dried in a critical point dryer, and mounted on stubs, according to Eisenback (1985). Afterward, nematodes were examined using an FEI Quanta 400 (Scanning Electron Microscope), operating at 20.0 kV and used in high vacuum mode.

### Molecular and phylogenetic analyses

Three adult nematodes extracted from palm fibers and three adult nematodes extracted from five *P. archon* larvae of the four palms were individually placed in a 0.2 ml tube (72 nematodes in total) containing: 50.0 μl InstaGene Matrix (BioRad), 0.5% SDS, and 2.5 μl Proteinase K 20.0 μg/μl (QIAGEN), and subsequently incubated at 55.0°C for 3 h. DNA was recovered through alcoholic precipitation performed with 100 μl of cold absolute ethanol and resuspended in 25 μl of double distilled sterile water.

A sequence of 18S-ITS-28S locus was obtained through assembly of five overlapping PCR amplicons: the SSU gene was sequenced using three primers pair 988F (Holterman et al., 2006) (5′ – CTCAAAGATTAAGCCATGC – 3′) / 1912R (Holterman et al., 2006) (5′ – TTTACGGTCAGAACTAGGG – 3′) [annealing temperature Ta = 45°C], 1813F (Holterman et al., 2006) (5′ – CTGCGTGAGAGGTGAAAT – 3′) / 2646R (Holterman et al., 2006) (5′ – GCTACCTTGTTACGACTTTT – 3′) [Ta = 45°C] and nem1 (Foucher and Wilson, 2002) (5′ – GCAAGTCTGGTGCCAGCAGC – 3′) / nem2 (Foucher and Wilson, 2002) (5′ – CCGTGTTGAGTCAAATTAAG – 3′) [Touchdown PCR Ta from 55°C to 45°C with a ΔTa −1°C/cyle]; ITS region using 18S (Vrain et al., 1992) (5′ – TTGATTACGTCCCTGCCCTTT – 3′) / 28Srev430 (Skoracka et al., 2014) (5′ – CAACTTTCCCTCACGGTACTTGT – 3′) [Ta = 55°C] and LSU D2–D3 region using D2F (Nguyen et al., 2004) (5′ – CCTTAGTAACGGCGAGTGAAA – 3′) / D3B (De Ley et al., 2005) (5′ – TCGGAAGGAACCAGCTACTA – 3′) [Ta = 55°C]. Each endpoint PCR reaction was performed in total volume of 50 μl containing 25 μl 2X DreamTaq Hot Start PCR Master Mix (Thermo Fisher), 0.6 μM each primer, and 5.0 μl extracted DNA. The thermal protocol was adopted as follows: an initial denaturation step for 3 min at 95°C followed by 45 cycles of denaturation at 94°C for 60 s, primer annealing temperature (Ta) for 60 s, elongation at 72°C for 1.5 min, followed by a final elongation step of 5 min at 72°C. Amplicons were purified and sequenced “in house” on SeqStudio sequencer (Thermo Fisher). The resulting sequences were assembled using Geneious Prime v. 2023.0.3 (Biomatters Ltd.) and submitted to GenBank.

Phylogenetic analyses were conducted using 18S (SSU) and 28S (LSU) gene fragments; sequences from orthologous loci belonging to other *Rhabditis* specimens were mined from GenBank. The two final datasets were 874 and 280 positions long and included 155 and 32 sequences for 18S and 28S gene fragments, respectively. The choice of the most suitable substitution matrixes was evaluated using jModelTest2 (Darriba et al., 2012) using BIC criteria, while the phylogenetic tree was inferred by Bayesian approach using BEAST2 v2.7.6 (Bouckaert et al., 2019) assuming TMV+G (4 categories) and GTR+G (4 categories) as nucleotide substitution matrixes, under Yule coalescent demographic model. The MCMC chain was run for 1 million generations, discarding the first 10% as burn-in. Species delimitation analysis was performed with the BEAST2 package Speedemon (Douglas and Bouckaert, 2022). The phylogenetic reconstructions were obtained from five independent MCMC runs resuming the obtained trees at 95% HDP.

### Pathogenicity assays

Extracted nematodes were used to assess their pathogenicity against the insect model *Galleria mellonella* (Lepidoptera: Pyralidae). The experimental unit consisted of a Petri dish (3.5 cm diameter) with two sheets of filter paper and a *G. mellonella* larva. A tap water suspension of 250 μL containing approximately 300 individuals of different stages (males, females, and juveniles) was inoculated into each Petri dish. In the control, only tap water was added. The experimental units were stored in the dark at 23±1 °C for one week. Larval mortality was checked daily. Dead larvae were individually placed on modified White traps and observed daily to recover the emerging nematodes. The experiment was repeated twice (n=10 treated with nematodes and n=10 as control for each repetition).

## Results and Discussion

The nematode *Rhabditis (Rhabditella) axei* was extracted from all palm fibers infested by the alien invasive insect *Paysandisia archon* and from the dead larvae found in the palm trunks. In the first palm collection site, nematodes emerged from only one dead *P. archon* larva. In the second palm, four dead larvae showed the presence of nematodes. In the third and fourth palm, nematodes were present in all dead larvae.

Nematodes were detected in the feces of all examined alive larvae as well as in the feces of the mollusk recovered in the fourth palm. In the dissected larvae, nematodes were found also in the gut content. A mix of nematode stages (juveniles, male and female adults) emerged from all the types of samples analyzed: palm fibers, decomposing *P. archon* larvae, feces, and gut content.

**Figure 1:**
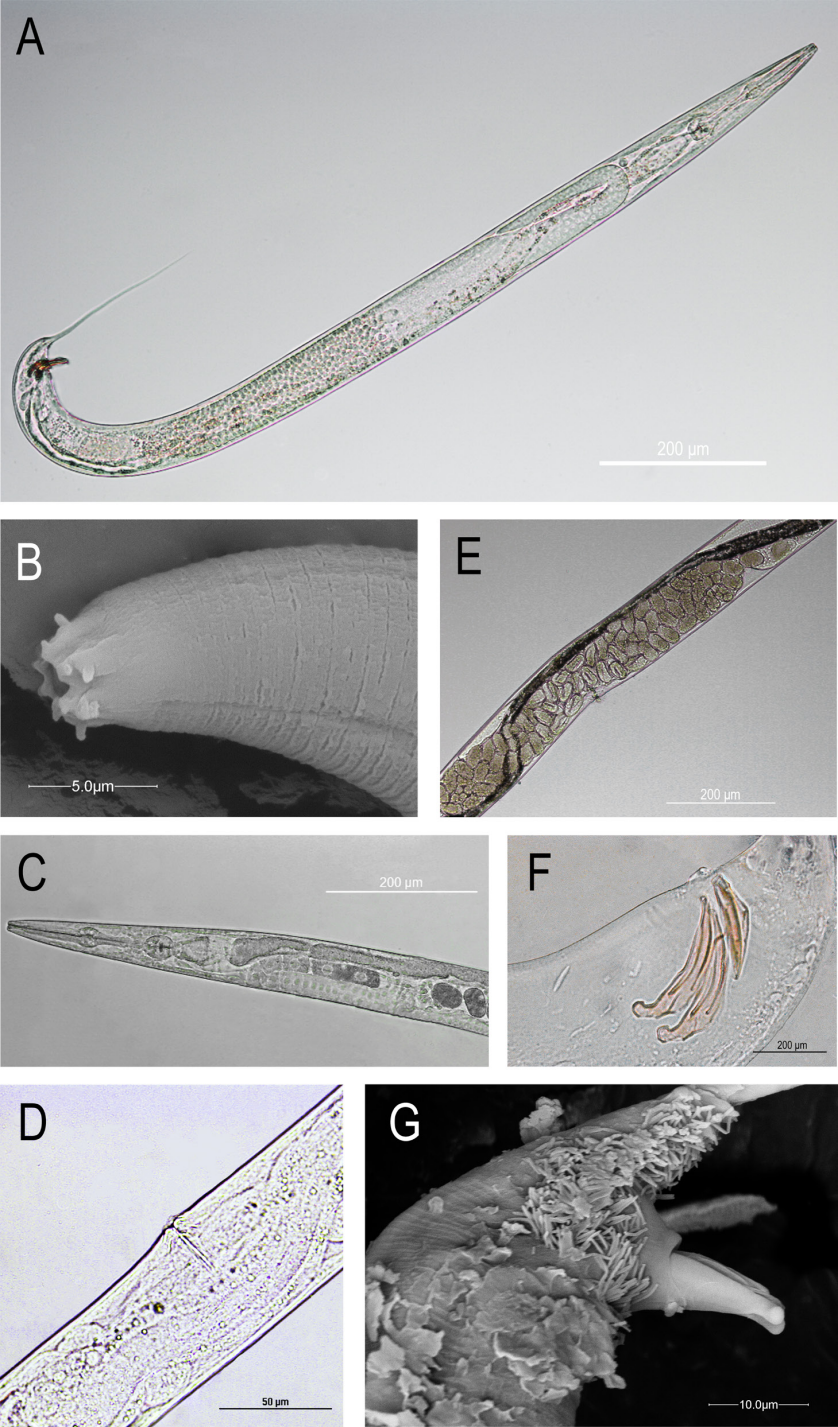
Rhabditis *axei*: (A) male, entire body; (B) head region at SEM; (C) pharyngeal region; (D) vulva; (E) eggs at mid-body; (F) spicules and gubernaculum; (G) spicules at SEM.

To identify the collected nematodes, specimens were characterized by morphological, morphometrical, and molecular analyses. At microscopic observation, the specimens are vermiform (Figure 1-A). A narrow buccal cavity presents a distinctive lip region composed of six partially fused lips slightly cone shaped. Bristle-like labial sensilla are present on the lips. The apical part of the lips is smooth without any striated pattern. The low median part of the lips is characterized by a cuticle with fine annulated pattern which appears more marked as it gets far from the buccal cavity (Figure 1-B). Stoma length ranged from 23.4 to 34.4 μm in females, and from 21.9 to 30.9 μm in males. Rhabditiform oesophagus (female range: 191.8 – 213.2 μm; male range: 183.8 – 233.5 μm) presents a valved terminal bulb. (Figures 1-A, 1-C). Reproductive system of the females is didelphic, with vulva in form of a transverse slit located at mid-body (Figure 1-D). Embryonated eggs (35.0 – 60.1 μm) could be seen in uteri (Figure 1-E). The tail is long and conical. Males are morphologically similar to females except for the reproductive system and tail shape. Spicules are paired, equal in length (37.7–53.9 μm), and separated by a double groove on the lateral surface. The proximal edge of the spicules is characterized by a wide manubrium and ends in a rounded tip (Figures 1-F, 1-G), gubernaculum (26.0 – 35.4 μm) is dorsal to spicules, that ranged from 37.7–53.9 μm. The tail is long and filamentous (Figure 1-A) (See Table 1).

**Table 1.** Measurements Morphometrics of Rhabditis axei (14 females and 21 males). All measurements are in μm: mean ± s.d. (range). Comparison with measurements given by Goodey (1963) and Meamar et al. (2007). L: body length; MBD: maximum body diameter; ES: distance of the base of the pharynx from head end; B: buccal cavity length; T: tail length; SP: spicule length; GU: gubernaculum length; V: distance of vulva from head end; a: L/MBD; b: L/ES; c: L/T; V%: V/L*100.

**Character**	**Current study**		**According to Goodey, 1963**		**According to Meamar et al., 2007**	
		
	**Female (μm)**	**Male (μm)**	**Female (μm)**	**Male (μm)**	**Female (μm)**	**Male (μm)**
L	1369.1 ± 196.1 (1128.2–1862.4)	1236.5 ± 194.9 (890.9–1705.0)	(760.0–1180.0)	(740.0–950.0)	1135.7 ± 88.3 (910.0–1374.1)	894.3 ± 62.7 (673.4–1137.5)
MBD	63.4 ± 14.9 (42.5–92.6)	47.2 ± 13.9 (25.4–75.9)	-	-	53.7 ± 5.3 (45.5–68.2)	39.9 ± 3.3 (27.3–54.6)
ES	213.2 ± 17.1 (191.8–242.8)	205.2 ± 11.8 (183.8–233.5)	-	-	-	-
B	26.5 ± 3.1 (23.4–34.4)	24.6 ± 2.1 (21.9–30.9)	-	-	22.5 ± 1.4 (19.9–26.4)	22.9 ± 1.2 (19.8–26.4)
T	301.7 ± 56.3 (221.5–434.4)	265.8 ± 38.6 (177.1–332.8)	-	-	307.6 ± 25.8 (235.4–363.0)	189.1 ± 12.8 (154.0–228.8)
SP	-	44.5 ± 5.0 (37.7–53.9)	-	-	-	38.1 ± 1.3 (33.0–44.0)
GU	-	30.2 ± 3.1 (26.0–35.4)	-	-	-	27.3 ± 0.9 (24.2–30.8)
V	664.9 ± 99.1 (524.6 – 912.4)	-	-	-	490.9 ± 39.6 451.4–530.5	-
a	22.2 ± 3.5 (16.8–29.4)	27.4 ± 5.4 (18.7–39.6)	(20.0–32.0)	-	-	(21.0–29.0)
b	6.4 ± 0.8 (4.8–8.2)	6.0 ± 0.8 (4.5–7.6)	(4.1–6.5)	-	-	(4.0–5.4)
c	4.6 ± 0.5 (3.8–5.4)	4.6 ± 0.6 (3.8–7.0)	(3.6–5.0)	-	-	(3.5–5.0)
V%	48.6 ± 2.8 (44.8–53.8)	-	(38.0–50.0)	-	-	-

Morphological features and morphometric measurements identified the nematodes as *Rhabditis (Rhabditella) axei* (Cobbold, 1884; Chitwood, 1933). However, the specimens found in this survey showed several differences compared to those reported by Goodey (1963) and Meamar et al. (2007). The sizes of our specimens of *R. axei* are larger for the following characters: body length, maximum body diameter, buccal cavity length, and distance of vulva from head end for the females, and body length, maximum body diameter, buccal cavity length, tail length, spicule length, and gubernaculum length for males (see Table 1).

The several occurrences of this nematode in independent collection sites raise the question of its source and how it was transmitted to the different palms infested by *P. archon*. The nematodes could be transported by mollusks present in the infested palms, since, in our study, we also found nematodes in mollusk feces, or by *P. archon* adults or larvae of Diptera, but this last issue needs to be investigated in the future. In fact, Kiontke (1999) reported that in some species of *Rhabditis* (*Rhabditella*), the dauer juveniles are phoretic and transported by insects, but no data are available for *R. axei*.

A haplotypic analysis of the collected nematodes was performed on a large genetic locus of rDNA's genetic region. The resulting assembly of the PCR products gave a DNA contig 3.3 Kb long that covers the rDNA locus from the 5′ region of the 18S gene to the D2–D3 loop region of the 28S gene. Sequences were submitted to GenBank with the progressive accession numbers: PP135622 “Impruneta”, PP135623 “Firenze-Isolotto”, and PP135624 “Firenze-Cascine del Riccio”. The sequences of the nematodes extracted from the two palms of “Impruneta” were identical. Sequence comparison identified three distinct haplotypes that differ in five polymorphic sites in total; all nematodes isolated from the same sampling site share the same haplotype. Homology searches in the GenBank database using the 18S, ITS, and 28S consensus regions as queries identified the samples as *Rhabditis* (*Rhabditella*) *axei* (locus 18S: 99.3%ID in 1611 positions with *Rhabditella axei* isolate DF5006 [GenBank U13934]; locus ITS: 96.9%ID in 613 positions with *Rhabditella axei* [GenBank DQ121441]; locus 28S D2–D3 region: 100%ID in 818 positions with *Rhabditella axei* isolate DF5006 [GenBank AY602177]).

Phylogenetic reconstructions based on the 28S gene provide some insight into the variability of the *Rhabditis* genus. All nine species considered, except *R. terricola,* were monophyletic, and recent nodes were generally well supported with posterior probability values between 0.9 and 1. Species delimitation analysis identified *Rhabditis* sp. CM-2010 as the closest species to *R. axei*, and the probability of correct identification of a sample using a BLAST homology search (or placement on a phylogenetic tree) given the same database was 0.98 (acceptance limits ranging from 0.87 to 1.0).

Due to the scarcity in GenBank of *Rhabditis* species characterized with the same region of this target (only 5 species, but more than 150 sequences of *Rhabditis* cf. *terricola*), less data can be obtained from phylogenetic inference based on the 18S gene. This gene showed a lower intraspecific variability in *R. axei* compared to 28S (0.005 for 18S compared to 0.008 for 28S).

As regards the pathogenicity, the test carried out on *G. mellonella* larvae showed no entomopathogenic effects, since only two larvae in the control and two in the treatment were found dead after 72 hours. The other larvae were alive after one week. Furthermore, no nematodes emerged from the cadavers placed in the White traps.

In this report, we described the first and recurrent association of *Rhabditis* (*Rhabditella*) *axei* from different sources, such as *T. fortunei* palm fibers, and live and dead larvae of the *Paysandisia archon* in three independent sites in Tuscany (Central Italy). Based on our data, we can therefore exclude the entomopathogenic and parasitic behavior of this species, at least against Lepidoptera. In this context, *Rhabditis* (*Rhabditella*) *axei* is 1) free-living since it was extracted from palm fibers; 2) pseudoparasitic (Pierre et al., 1995); 3) phoretic (Dillman et al, 2012) since it was found in the gut contents of live larvae without causing damage and can use the host for transport; and 4) necromenic (Dillman et al., 2012) since we observed the multiplication of this nematode in the decaying larvae of *P. archon* without being involved in the insect death.

## Figures and Tables

**Figure S1:**
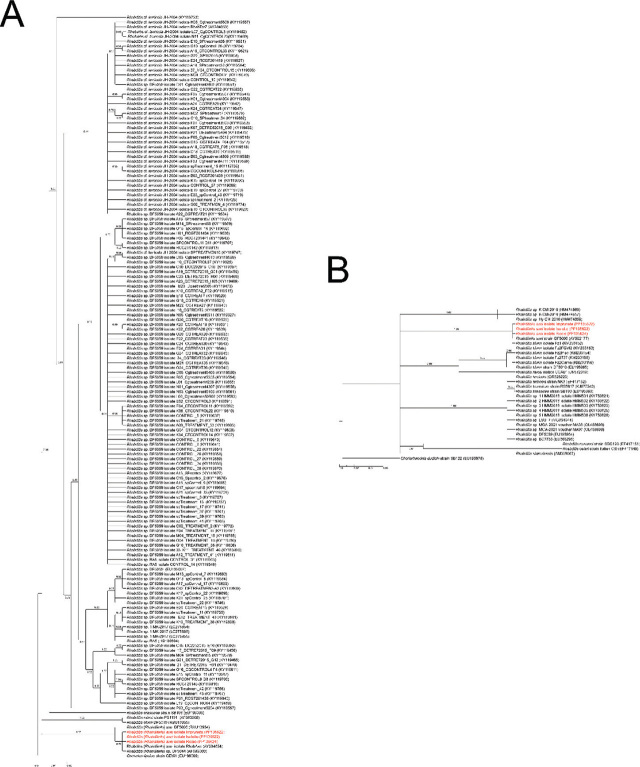
Bayesian trees of the genus Rhabditis based on A) SSU and B) LSU gene fragments. Accession numbers of sequences were reported in brackets and posterior probability was annotated near the branches. The strains isolated during this work were highlighted in red.
